# Synergistic mechanisms of DGAT and PDAT in shaping triacylglycerol diversity: evolutionary insights and metabolic engineering strategies

**DOI:** 10.3389/fpls.2025.1598815

**Published:** 2025-07-01

**Authors:** Wen-Lu Cai, Shui-Yan Yu, Yong-Hong Hu

**Affiliations:** ^1^ Shanghai Key Laboratory of Plant Functional Genomics and Resources, Shanghai Chenshan Botanical Garden, Shanghai, China; ^2^ CAS Center for Excellence in Molecular Plant Sciences, University of the Chinese Academy of Sciences, Beijing, China

**Keywords:** diacylglycerol acyltransferase (DGAT), phospholipid:diacylglycerol acyltransferase (PDAT), triacylglycerol, fatty acid profiles, plant oils

## Abstract

Triacylglycerol (TAG), the primary storage lipid in plants, determines oil quality through its fatty acid composition. This review focuses on the biosynthesis of TAG, systematically analyzing the mechanistic similarities and differences between the acyl-CoA-dependent Kennedy pathway (catalyzed by the rate-limiting enzyme DGAT) and the acyl-CoA-independent pathway (regulated by the rate-limiting enzyme PDAT). By integrating functional studies, evolutionary analyses, and lipidomic data, we reveal the distinct substrate preferences of DGAT and PDAT, their differential contributions to TAG synthesis, and their synergistic mechanisms in shaping triacylglycerol diversity. This work establishes a theoretical framework for the targeted engineering of plant oils with enhanced nutritional and industrial value through optimized fatty acid profiles.

## Introduction

Plant oils are essential for human daily life, providing the necessary nutrients for normal physiological development (Khalili Tilami and Kouřimská, 2022; Wei et al., 2022; He et al., 2023), and serve as important industrial raw materials and energy resources (Absalome et al., 2020; Mishra et al., 2024). Triacylglycerol (TAG), the primary storage lipid component of mature seeds, is the metabolic product of fatty acid synthesis (Zhu et al., 2012). Structurally, TAG consists of one glycerol molecule and three fatty acid molecules. Although the glycerol backbone has a simple molecular structure, the chain length and the number and positions of double bonds in fatty acids vary significantly. Consequently, the fatty acid type and relative content determine the quality and commercial value of storage oils. The application of lipidomics tools enables the determination of fatty acids composition in seed TAGs (Woodfield et al., 2018; Abdelghany et al., 2020; Yu et al., 2021; Zeng et al., 2024). Most plant oils contain five common fatty acids: palmitic (C16:0), stearic (C18:0), oleic acid (C18:1^Δ9^), linoleic acid (C18:2^Δ9,12^), and α-linolenic acids(C18:3^Δ9,12,15^). Among them, palmitic and stearic acids are saturated fatty acids. Oleic acid is a monounsaturated fatty acid (MUFA), and linoleic and α-linolenic acids (PUFA) are polyunsaturated fatty acids. Oleic acid is the major component of many edible oils, with a higher antioxidative stability and a better shelf life than polyunsaturated fatty acids. Studies have shown that MUFAs can lower cholesterol levels and reduce the risk of heart disease (Gillingham et al., 2011). Furthermore, they have anti-aging and anti-inflammatory effects, which makes them suitable raw materials for cosmetic products (Uitterhaegen et al., 2016). Linoleic and α-linolenic acids, which are essential fatty acids for humans and mammals, cannot be synthesized by their bodies and must be obtained through diet. Once absorbed, they are further metabolized into other nutrients and have functions such as brain development promotion, retinal function improvement, inflammation reduction, blood pressure reduction, and cardiovascular disease prevention (Wallis et al., 2002; Swanson et al., 2012; Mocking et al., 2016; Gorusupudi et al., 2021). Despite being vital nutrients for human health, PUFAs are prone to oxidation, have weak stability, and are not well suited for storage, making them unsuitable as biofuels or raw chemical materials (Kodali, 2002).

TAG synthesis occurs via the acyl-CoA-dependent Kennedy and the acyl-CoA-independent pathways, with interspecies differences. Some species exclusively utilize the Kennedy pathway to synthesize TAG, while in others (e.g., soybeans), over 90% of TAG is formed through the acyl-CoA-independent pathway (Bates, 2016). These pathways involve distinct acyltransferases: glycerol-3-phosphate acyltransferase (GPAT), lysophosphatidic acid acyltransferase (LPAAT), and diacylglycerol acyltransferase (DGAT) in the Kennedy pathway and phospholipid acyltransferase (PDAT) in the acyl-CoA-independent pathway. These enzymes exhibit different substrate preferences for fatty acids, which vary by species and influence the fatty acid composition and content of seed storage oils.

This review summarizes differences in fatty acid composition and content between traditional and novel oil crops, focusing on the structural, functional, and regulatory aspects of two enzyme types, DGAT and PDAT, involved in TAG synthesis, with an emphasis on their preferences for fatty acid substrates. By comparing the differences in fatty acid selection for TAG synthesis at the *sn*-3 position between DGAT and PDAT, potential targets can be identified for the directional design and precise breeding of new oil crops. This will provide theoretical and data support for rational and efficient resource utilization, contributing to economic development and improved living standards.

## Fatty acids composition differences in seed plants

Using Plant FA database resources (http://plantfadb.org/), data on the fatty acid composition and content of 7 traditional oilseed crops and 22 novel oilseed crops were collected. Through comparative analysis, it was found that there were differences in the types of dominant fatty acids contained in different crops. In traditional oil crops, such as corn (*Zea mays* L.), soybean (*Glycine max* L.), and peanut (*Arachis hypogaea* L.), oleic acid and linoleic acid dominate the fatty acid composition, but their contents vary among crops. For example, linoleic acid is the primary fatty acid component in corn, soybean, sesame (*Sesamum indicum* L.), and sunflower (*Helianthus annuus* L.), with relative contents approaching or exceeding 50%. In peanut and rapeseed (*Brassica napus* L.), the oleic acid content is the highest (**Table 1**). In recent years, certain novel oil crops have drawn increasing attention due to the significance of their fatty acid composition for human health and industrial applications. For instance, Buglossoides arvensis (*Lithospermum arvense*), perilla (*Perilla frutescens* (L.) Britt), and tree peony (*Paeonia suffruticosa* Andr.) are rich in α-linolenic acid, a member of the ω-3 fatty acid family. Although some novel oil crops, such as Barbados nut (Jatropha curcas L.), long-stem almonds (Amygdalus pedunculata Pall), and safflower (Carthamus tinctorius L.), primarily consist of oleic and linoleic acids, with their combined composition often exceeding 50% (**Table 1**), these crops also contain a variety of other important specialized fatty acid components. Barbados Nut, in particular, can yield sulfur-free clean diesel in large quantities, highlighting its significant industrial application value. Both Yellowhorn trees (*Xanthoceras sorbifolium*) and safflower contain a long-chain monounsaturated fatty acid called nervonic acid (C24:1^Δ15^) that promotes brain development. Niche plant oils, such as castor (*Ricinus communis* L.) and tung oil (*Vernicia fordii* Hemsl), are rich in special fatty acids, such as ricinoleic acid (accounting for about 89% of the total fatty acids in castor) and eleostearic acid (accounting for about 85% of the total fatty acids in tung) (Dyer et al., 2008; Park et al., 2008). Castor oil can carry out many chemical reactions through its hydroxyl group, double bond and carboxyl group (Ogunniyi, 2006), and the resulting formation is widely used in the coating industry (R et al., 2021), metal industry (R et al., 2021) and machinery industry (Guo et al., 2017). Tung oil is renowned for its exceptional technical properties, including superior waterproofing capabilities, rapid-drying characteristics, and remarkable corrosion inhibition (Karak, 2012). These unique physicochemical attributes have enabled its extensive applications across diverse industrial sectors, particularly in a drying component in paints, varnishes, coatings, and finishes (Zhang et al., 2014). It is also employed in synthesizing thermosetting polymers and resins with superior performance (Huang et al., 2013; Liu et al., 2016) and has been proposed as a potential source for bio-based diesel fuel (Park et al., 2008; Chen et al., 2012b; Shang et al., 2012).

**Table 1:** Composition of fatty acids in traditional and novel oil crops.

Common name	Latin name	Familia	Genus	Relative content of major fatty acids (%)					Special fatty acids
				C16:0	C18:0	C18:1^Δ9^	C18:2^Δ9,12^	C18:3^Δ9,12,15^	
Maize ^a^	Zea mays L.	Poaceae	Zea	12.3	1.9	27.7	56.1	1	—
Soybean ^a^	Glycine max L.	Fabaceae	Glycine	10.8	3.9	23.9	52.1	7.8	—
Sesame ^a^	Sesamum indicum L.	Pedaliaceae	Sesamum	7.83	5.41	27.22	48.99	0.01	C17:0; C17:1
Peanut ^a^	Arachis hypogaea L.	Fabaceae	Arachis	10.04	2.92	47.08	32.29	1.8	—
Sunflower ^a^	Helianthus annuus L.	Asteraceae	Helianthus	7.9	4.13	14.42	73.55	0	—
Rapeseed ^a^	Brassica napus L.	Brassicaceae	Brassica	5.1	1.7	60.1	21.5	9.9	—
Flax ^a^	Linum usitatissimum L.	Linaceae	Linum	6.1	3.4	18.4	16.8	55	—
Tung tree ^b^	Vernicia fordii (Hemsl.) Airy Shaw	Euphorbiaceae	Vernicia	2.68	2.42	6.35	8.19	80.03	C18:3,c9,t11,t13
Perilya ^b^	Perilla frutescens L. Britt.	Lamiaceae	Perilla	7.32	1.89	2.77	10.54	77.58	C17:0; C17:1
Buglossoides arvensis ^b^	Lithospermum arvense	Boraginaceae	Lithospermum	5	2	11	14	57	C18:4
Tree peony ^b^	Paeonia suffruticosa	Paeoniaceae	Paeonia	5.7	2.2	20.8	24.6	45.2	C17:0; C17:1
Chinese tallow tree ^b^	Sapium sebiferum L. Roxb.	Euphorbiaceae	Sapium	7.08	2.17	14.2	30.3	43.5	—
False flax ^b^	Camelina sativa L. Crantz	Brassicaceae	Camelina	5.04	2.15	15.65	18.88	38.15	C20:1
Sea buckthorn ^b^	Hippophae rhamnoides L.	Elaeagnaceae	Hippophae	27	1.4	21.7	15.7	8.8	—
English walnut ^b^	Juglans regia L.	Juglandaceae	Juglans	4.6	0.9	17.8	73.4	3.3	—
Chinese pistache ^b^	Pistacia chinensis Bunge	Anacardiaceae	Pistacia	18.55	0	47.59	32.35	0.98	—
Olive oil ^b^	Olea europaea L.	Oleaceae	Olea	8.76	2.83	72.79	13.2	0.92	—
Tea oil camellia ^b^	Camellia oleifera Abel	Theaceae	Camellia	11.7	1.8	75.1	10.5	0.9	—
Yellow nutsedge ^b^	Cyperus esculentus L.	Cyperaceae	Cyperus	16.2	2.5	64.9	15.5	0.9	—
Yellowhorn trees ^b^	Xanthoceras sorbifolium Bunge	Xanthoceraceae	Xanthoceras	5.3	1.92	30.69	42.44	0.41	—
African oil palm ^b^	Elaeis guineensis Jacq.	Arecaceae	Elaeis	43.8	4.4	39.1	10.2	0.3	—
Safflower ^b^	Carthamus tinctorius L.	Asteraceae	Carthamus	6.1	2.3	13.4	76	0.3	C20:5
Almonds with long stems ^b^	Amygdalus pedunculata Pall.	Rosaceae	Amygdalus	2.68	0.35	69.5	27.5	0.18	—
Barbados Nut ^b^	Jatropha curcas L.	Euphorbiaceae	Jatropha	27	3.2	69	0	0	—
Cotton ^b^	Gossypium sp.	Malvaceae	Gossypium	22.6	2.1	17.7	56.5	0	—
Chinese windmill palm ^b^	Trachycarpus fortunei H. Wendl.	Arecaceae	Trachycarpus	11	4.4	15.9	2.8	0	C12:0; C14:0
Coconut palm ^b^	Cocos nucifera L.	Arecaceae	Cocos	4.2	3	11.9	3.5	0	C10:0; C12:0
Idesia ^b^	Idesia polycarpa Maxim.	Salicaceae	Idesia	8.4	3.48	5.85	80.11	0	C18:3,c9,t11,t13
Castor bean ^b^	Ricinus communis L.	Euphorbiaceae	Ricinus	1.1	0.9	3.4	4.9	0	C18:1,12-OH-c9

a represents traditional oilseed crops, and b represents novel oilseed crops. Traditional oilseed crops refer to a category of plants that have been utilized by humans for an extended period. These crops possess a significant cultivation scale and contain high levels of fat in their seeds, which is extracted for use as edible oil or as raw materials for industrial and pharmaceutical applications. Novel oilseed crops refer to crop species that have been newly discovered or cultivated over the past decade. These species exhibit high oil content and are suitable for oil extraction. In addition to diversifying the range of oil crops, they also provide enhanced options and resources for the edible oil market.

**Figure 1:**
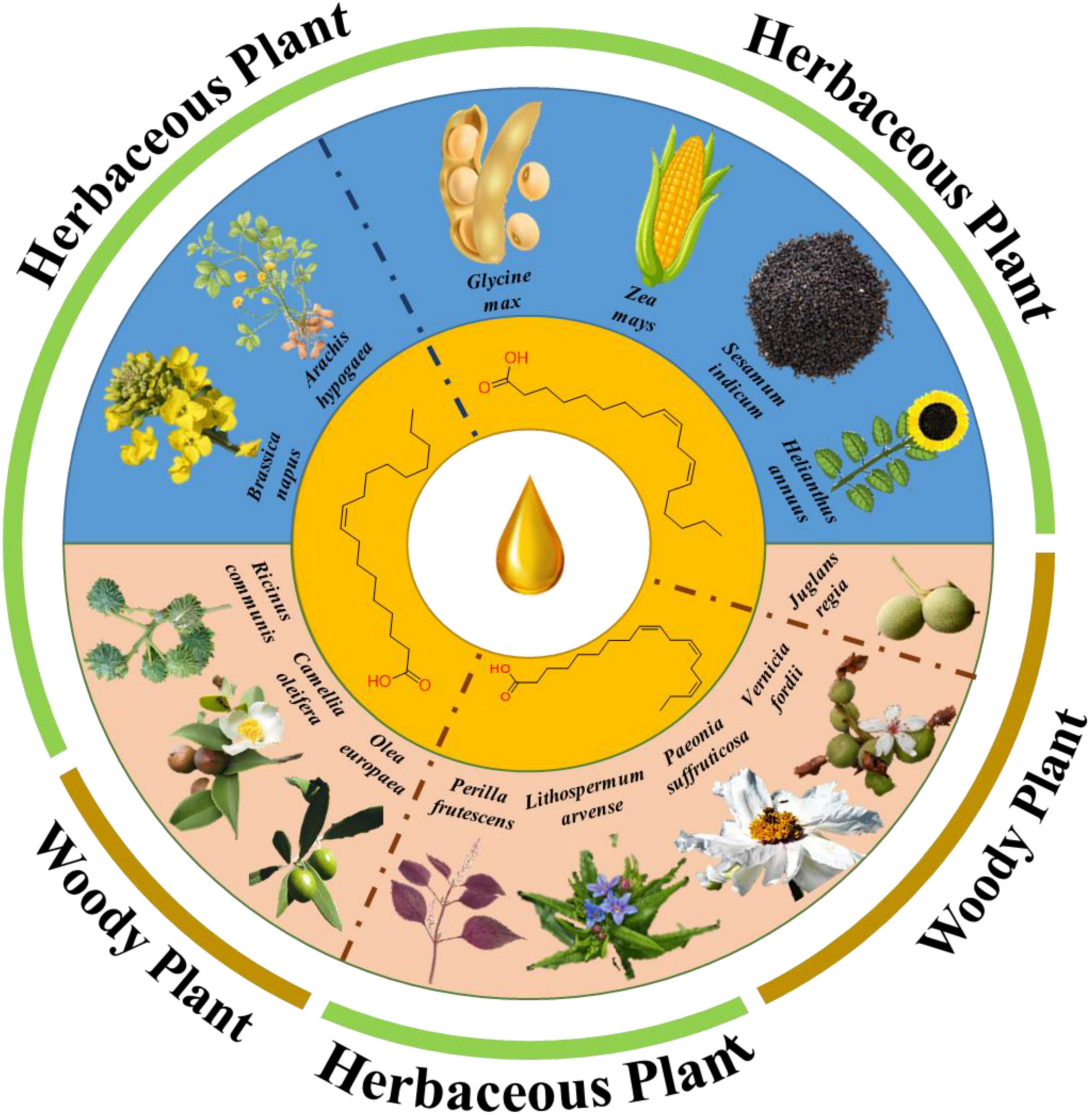
Predominant fatty acid types in selected oil crops. Blue represents traditional oil crops; Pink represents a novel oil crop.

In most oil crops, one or two fatty acids are dominant (**Figure 1**). Such a relatively singular fatty acid composition clashes with a diversified and complex dietary structure. Technological advances have led to the discovery of novel oil crops rich in specific polyunsaturated fatty acids. For example, tree peony contains odd-chain fatty acids (Yu et al., 2016), Chinese tallow tree (*Sapium sebiferum* (L.) Roxb.) produces 2,4-decadienoic acid (Ma et al., 2023), and false flax (*Camelina sativa* (L.)) Crantz) contains nervonic acid (Kukrić et al., 2022). Fatty acid metabolism is a primary metabolic process. Previous studies have shown that plant fatty acid synthesis, TAG assembly, and core enzyme-encoding genes exhibit a high degree of evolutionary conservation. However, variations in enzyme activity and concentration and substrate preferences are induced by different factors, which are regulated at various levels and directly affect the relative fatty acid content of seed oils.

## Pathways and regulation of triacylglycerol synthesis in plants

**Figure 2:**
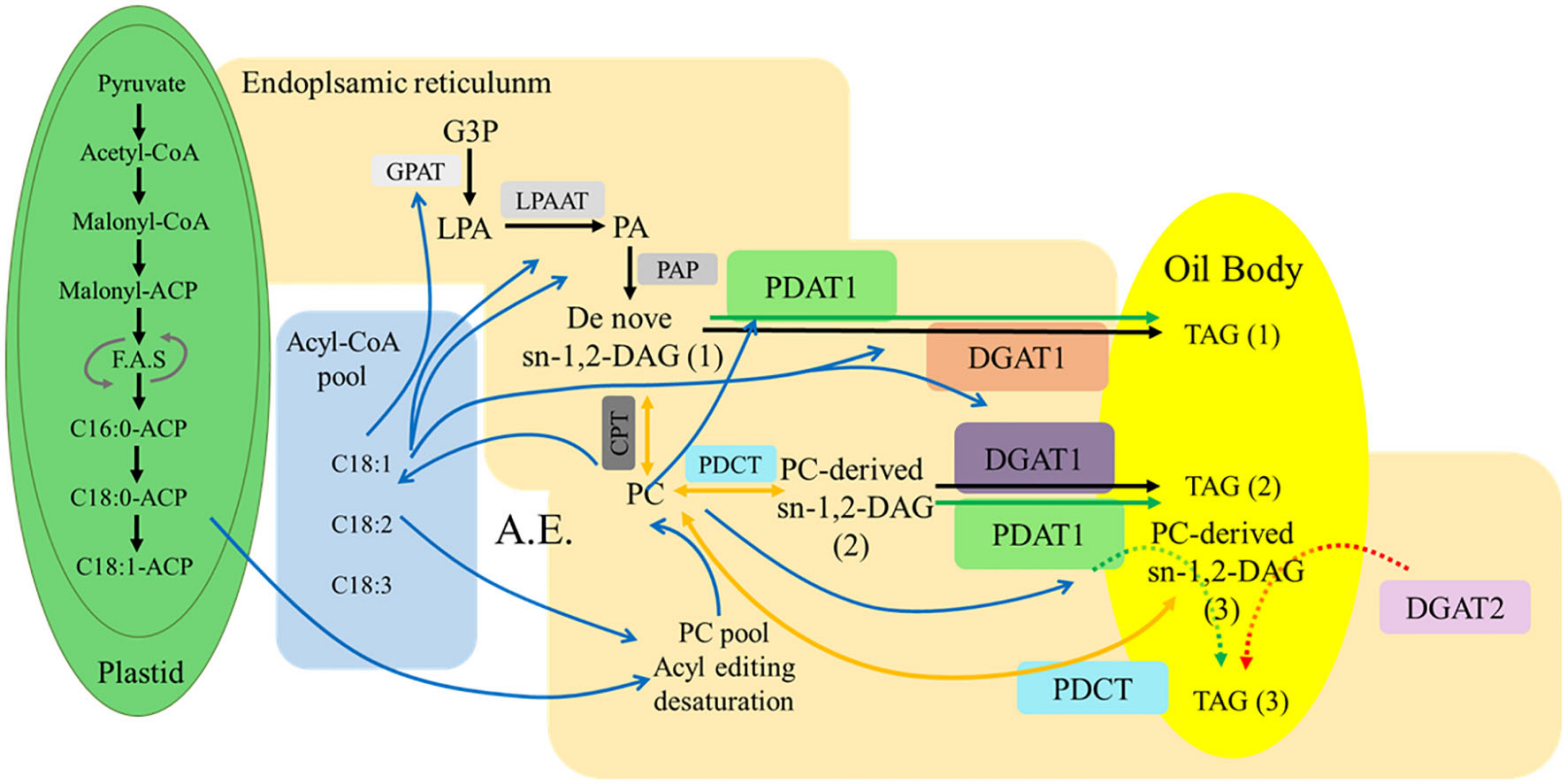
Fatty acid synthesis and triacylglycerol assembly pathways in plants. Blue lines indicate acyl flow direction. Black lines represent the Kennedy pathway. Yellow lines indicate the involvement of PC in the DAG synthesis pathway. Green arrows show TAG synthesis pathways in which PDAT participate. Red arrows represent TAG synthesis pathways involving DGAT2. The dashed lines indicate that the reactions occur less frequently in TAG synthesis pathway. Box with undercolor: Enzymes involved in TAG synthesis pathway. Acyl-CoA pool: elongation≥20C; PC pool: desaturation. Abbreviations: F.A.S: fatty acid synthesis; A.E.: Acyl editing; G3P: glycerol-3-phosphate; LPA: lysophosphatidic acid; PA: phosphatidic acid; DAG: diacylglycerol; PC: phosphatidylcholine; TAG: triacylglycerol; GPAT: glycerol-3-phosphate acyltransferase; LPAAT: lysophosphatidic acid acyltransferase; PAP: phosphatidic acid phosphatase; DGAT: acyl-CoA:diacylglycerol acyltransferase; PDAT: phospholipid:diacylglycerol acyltransferase; CPT: CDP-choline: diacylglycerol cholinephosphotransferase; PDCT: phosphatidylcholine: diacylglycerol cholinephosphotransferase. Image modified from Regmi et al., 2020 (doi: 10.1104/pp.20.00461).

TAG, as a major component of plant lipids, undergoes a biosynthetic process that can be divided into three steps (Xu et al., 2018). The first step involves raw material processing. In plastids and the cytoplasm, plants utilize glucose produced through photosynthesis to generate pyruvate and glyceraldehyde-3-phosphate via pathways such as glycolysis. Pyruvate is further processed into acetyl-CoA, which serves as a precursor for lipid synthesis. The second step is fatty acid chain synthesis and elongation. In plastids, acetyl-CoA carboxylase converts acyl-CoA into malonyl-CoA, which is subsequently catalyzed by fatty acid synthase to form acyl chains by sequentially adding two-carbon units from malonyl-CoA. These chains are transferred to the acyl carrier protein of fatty acid synthase. During this process, fatty acid chains are elongated to 16–18 carbons and undergo a single desaturation step before being released into the cytoplasm. The fatty acid composition exported from plastids in different plant species or distinct plant tissues is determined by the relative activities of acyl-ACP thioesterases and acyl-ACP desaturases (Lightner et al., 1994; Kachroo et al., 2007), which govern the production of various saturated fatty acids and monounsaturated fatty acids. The desaturation step occurring before the release of fatty acids from plastids primarily involves plastidial desaturases, such as FAD6 (Δ-6 desaturase) and FAD7/FAD8 (Δ-15 desaturase). These enzymes introduce double bonds into fatty acyl chains attached to monogalactosyldiacylglycerol (MGDG) and digalactosyldiacylglycerol (DGDG) within plastids, respectively. After export to the ER, further desaturation can occur, mediated by ER-localized enzymes like FAD2 (Δ12-desaturase) and FAD3 (Δ15-desaturase), which are critical for producing polyunsaturated fatty acids (PUFAs) such as linoleic acid and α-linolenic acid. These enzymes introduce double bonds into fatty acyl chains attached to phosphatidylglycerol (PG) or phosphatidylcholine (PC) within ER, respectively (Subedi et al., 2020). The final step involves fatty acid chain assembly onto a glycerol backbone to form TAG, which is facilitated by various acyltransferases (**Figure 2**).

In eukaryotic cells of higher plants and algae, TAG synthesis primarily occurs through two pathways: the acyl-CoA-dependent Kennedy and the acyl-CoA-independent pathways (**Figure 2**). The Kennedy pathway is catalyzed by a series of acyltransferases to form TAG through the acylation of glycerol-3-phosphate. The process begins with GPAT, which catalyzes the attachment of fatty acids from the acyl-CoA pool to the *sn*-1 position of glycerol-3-phosphate (Zheng et al., 2003). In the second step of the esterification reaction, LPAAT catalyzes the addition of fatty acids to the *sn*-2 position of the glycerol backbone (Yu et al., 2004). Before the third esterification step, phosphatidic acid phosphatase removes the phosphate group from the *sn*-3 position of the glycerol backbone, producing diacylglycerol. Finally, DGAT catalyzes the attachment of fatty acids from the acyl-CoA pool to the *sn*-3 position of diacylglycerol, forming TAG (1) (**Figure 2**).

The second TAG synthesis pathway is independent of acyl-CoA. Here, phospholipids, primarily phosphatidylcholine (PC) and phosphatidylethanolamine (PE), serve as acyl donors, and diacylglycerol acts as the acyl acceptor. Under PDAT’’s catalytic action, acyl groups are transferred from the *sn*-2 position of phospholipids to the *sn*-3 position of diacylglycerol, forming TAG (2) and lysophospholipids. Lysophospholipids can be further acylated using acyl groups from the acyl-CoA pool by lysophosphatidylcholine acyltransferase (**Figure 2**). This modified phospholipid is then used by PDAT to form TAG (2). PDAT-mediated synthesis of TAG is primarily observed in yeasts and vascular plants, with a preference for fatty acids modified via an acyl transfer reaction (Banaś et al., 2013). The primary difference between the Kennedy and PDAT pathways lies in the source of the acyl donor during the conversion of diacylglycerol to TAG. Kennedy directly uses acyl-CoA, while PDAT favors acyl groups from the *sn*-2 position of phospholipids.

Enzyme activity and abundance can be regulated at the DNA, RNA, and protein levels. Currently, research on DGAT and PDAT regulation in plants remains limited. In yeast, inactivation of the transcriptional activator Zap1p reduces mitochondrial pyruvate utilization, increasing LRO1 and PDAT expression, which results in TAG accumulation (Singh et al., 2016). This suggests a connection between *LRO1* expression and increased carbon flux towards lipid synthesis. At the protein level, LRO1 stability is controlled by ubiquitin ligase Hrd1 (Iwasa et al., 2016). In yeast mutants H1246, acyl-CoA-binding proteins (ACBP) from rapeseed have been shown to regulate DGAT1 activity (Sandager et al., 2002). When co-incubated with yeast microsomes of H1246 which expressed *Bn*DGAT1 and low concentrations of rBnACBP (ACBP:acyl-CoA ratio 0.33), DGAT activity was found to be increased by 20% (Yurchenko and Weselake, 2011). However, after continuing to add rBnACBP to the reaction system, the TAG content decreased (Yurchenko and Weselake, 2011). These findings indicate that TAG synthesis is not determined by a single enzymatic reaction but by a complex metabolic network. The complexity of TAG metabolism and regulation contributes to differences in fatty acid composition and content among oil crops.

Acyltransferases are widely present in plants, animals, and microorganisms. These enzymes transfer acyl groups from donors to acceptors (Bontpart et al., 2015). Currently, 20 acyltransferase types have been identified, and they utilize fatty acids or fatty alcohols as donors to form alkyl hydroxyl cinnamates (6 acyltransferase types are involved) or glycerides (12 acyltransferase types are involved) (Bontpart et al., 2015). The latter group primarily includes GPAT, LPAAT, DGAT, and PDAT, which use glycerol derivatives as acceptors and long-chain fatty acids as donors. GPAT catalyzes the acylation of the *sn*-1 position in TAG, producing lysophosphatidic acid (Jayawardhane et al., 2018). In *Arabidopsis*, 10 GPAT types have been identified and characterized, including ATS1 and GPAT1-9. ATS1 localizes to the plastid stroma, GPAT1–3 to the mitochondrial membrane, and GPAT4–9 to the ER membrane. Based on subcellular localization, GPAT in plants can be grouped into three classes, each contributing to the synthesis of distinct lipid categories, namely extracellular lipids, membrane lipids, and storage lipids (Bertrams and Heinz, 1981; Gidda et al., 2009; Chen et al., 2011; Yang et al., 2012; Singer et al., 2016). LPAAT is a crucial enzyme catalyzing the acylation of the *sn*-2 position of TAG and playing an essential role in converting lysophosphatidic acid to phosphatidic acid (Baud and Lepiniec, 2010; Misra et al., 2014). Several *LPAAT* genes have been cloned and characterized in plants, such as maize (Brown et al., 1994), *Arabidopsis* (Kim et al., 2005), nasturtium (*Tropaeolum majus*) (Taylor et al., 2010), peanut (Chen et al., 2012a), and castor (Arroyo-Caro et al., 2013).

## Identification and functional diversity of the DGAT gene family

The DGAT family has four subfamilies: DGAT1, DGAT2, DGAT3, and WS/DGAT (Kalscheuer and Steinbüchel, 2003; Hernández et al., 2012). Among these, *DGAT1* and *DGAT2* encode membrane proteins, and *DGAT3* encodes soluble proteins (Liu et al., 2012). DGAT enzymatic activity was first identified in chicken liver tissue in 1956 (Weiss et al., 1960), but the *DGAT1* gene was not cloned until 40 years later using a murine acyl-CoA:cholesterol acyltransferase sequence (Cases et al., 1998). There are species differences in the number of transmembrane domains of DGAT, mainly concentrated in eight to ten (Liu et al., 2012). For example, *EpDGAT1* is predicted to have 10 transmembrane domains (Mañas-Fernández et al., 2009). When predicting the structure of sunflower *DGAT1*, it was found that *HaDGAT1* had nine transmembrane structures (Sun et al., 2011). However, McFie et al. experimentally proved that the murine isoform had three transmembrane domains (McFie et al., 2010). In addition, they also found that although the N-terminal domain is not required for TAG synthesis, it is critical for enzyme activity and oligomer formation (McFie et al., 2010). For example, the DGAT1 amino acid sequence in *Kochia scoparia* shares 91% similarity with that in *Arabidopsis.* DGAT1 in *K. scoparia* exhibits acetyltransferase and long-chain fatty acid acyltransferase activities, which can switch depending on environmental conditions. This unique acetyltransferase activity is attributed to its special N-terminal structure (Milcamps et al., 2005). In seeds, DGAT1 is primarily associated with oil bodies and ER membranes (Gurr et al., 1974; Lacey and Hills, 1996). When DGAT1 is expressed in *Arabidopsis* leaves, its protein binds to the chloroplast membrane (Kaup et al., 2002). DGAT1 expression levels vary among plant tissues. In most higher plants, DGAT1 is expressed in all organs, with high expression levels in developing seeds, petals, and flower buds but low levels in leaves and stems (Hobbs et al., 1999). Expression patterns in dicots, such as soybean, vernonia (*Strobocalyx esculenta*), and rapeseed, are similar to those in *Arabidopsis* (Hobbs et al., 1999; Zhang et al., 2017; Volynets et al., 2022), whereas DGAT1 in nasturtium is exclusively expressed in developing seeds (Xu et al., 2008).

The existence of a second DGAT enzyme that plays a compensatory role in TAG synthesis was hypothesized after homozygous *DGAT1* knockout mice survived and synthesized TAG (Smith et al., 2000). Subsequently, *DGAT2* was purified and cloned from *Mortierella ramanniana*, DGAT2 participates in the bulk synthesis of TAG (Lardizabal et al., 2001). DGAT1 and DGAT2 exhibit different expression patterns depending on the cell type and perform non-redundant functions. Although DGAT1-deficient mice survive (Smith et al., 2000), homozygous DGAT2 knockout mice do not because of altered skin barriers and severely reduced systemic TAG levels (Stone et al., 2004). DGAT2 belongs to the acyl-CoA: monoacylglycerol acyltransferase family and differs from DGAT1 due to its shorter amino acid sequence and fewer transmembrane domains (Xu et al., 2018). In yeast, DGAT2 has four transmembrane domains, with both the N- and C-terminal domains functioning in the cytoplasm (Liu et al., 2011). Subcellular localization studies indicate that, similar to DGAT1, DGAT2 is found in oil bodies and ER membranes (Gurr et al., 1974; Lacey and Hills, 1996). *DGAT2* in tung tree is highly and specifically expressed in developing seeds and is associated with tri-eleostearin accumulation (Kroon et al., 2006; Shockey et al., 2006).

DGAT3, encoding a cytoplasm-localized soluble protein, was first identified in peanut (Saha et al., 2006). Studies have shown that DGAT3 is highly and specifically expressed in peanut seeds 8–24 days after flowering, but its expression decreases after 25 days and is absent during late seed development as well as in roots and leaves. Phylogenetic analysis of DGATs in Perilla (Xu et al., 2022), soybean (Zhao et al., 2019), oil palm (Rosli et al., 2018), and maize (Yan et al., 2018) has shown that the DGAT1/2/3 subfamily genes are conserved in plants. As research continues, the understanding of DGATs’ functions has become more comprehensive. For example, heterologous expression of the five soybean DGAT genes, *GmDGAT1A*, *GmDGAT1B*, *GmDGAT1C*, *GmDGAT2A*, and *GmDGAT2B* in *Arabidopsis* demonstrated that constitutive *GmDGAT1A* and *GmDGAT1B* expression reduced the seed protein content and increased the oil content (Zhao et al., 2019). GmDGAT1A overexpression increased the weight of individual seeds without affecting the total seed yield per plant, revealing that soybean DGATs can enhance oil synthesis in transgenic *Arabidopsis* seeds (Zhao et al., 2019). Similarly, *PrDGAT3* overexpression from *Paeonia rockii* in *Nicotiana benthamiana* leaf tissues and *Arabidopsis* significantly increased the TAG content (Han et al., 2022). Functional validation of DGATs can be achieved through heterologous and homologous expression. In the woody plant physic nut, overexpression of its own *JcDGAT1* and *JcDGAT2* genes significantly increased seed oil yield to 53.7 and 55.7% of the seed dry weight, respectively (Zhang et al., 2021). Additionally, the TAG content in the leaves of transgenic plants was 2–4 times higher than that in control plants. Although most research suggests a close relationship between DGATs and lipid synthesis, some studies indicate that DGATs function beyond lipid biosynthesis. For instance, the castor enzyme RcDGAT3 could not restore TAG biosynthesis in a yeast mutant but exhibited greater tolerance to free fatty acids compared with other DGAT enzymes, suggesting that RcDGAT3 has additional cellular functions (Trenz et al., 2023).

## Identification and functional differences in the PDAT gene family

**Figure 3:**
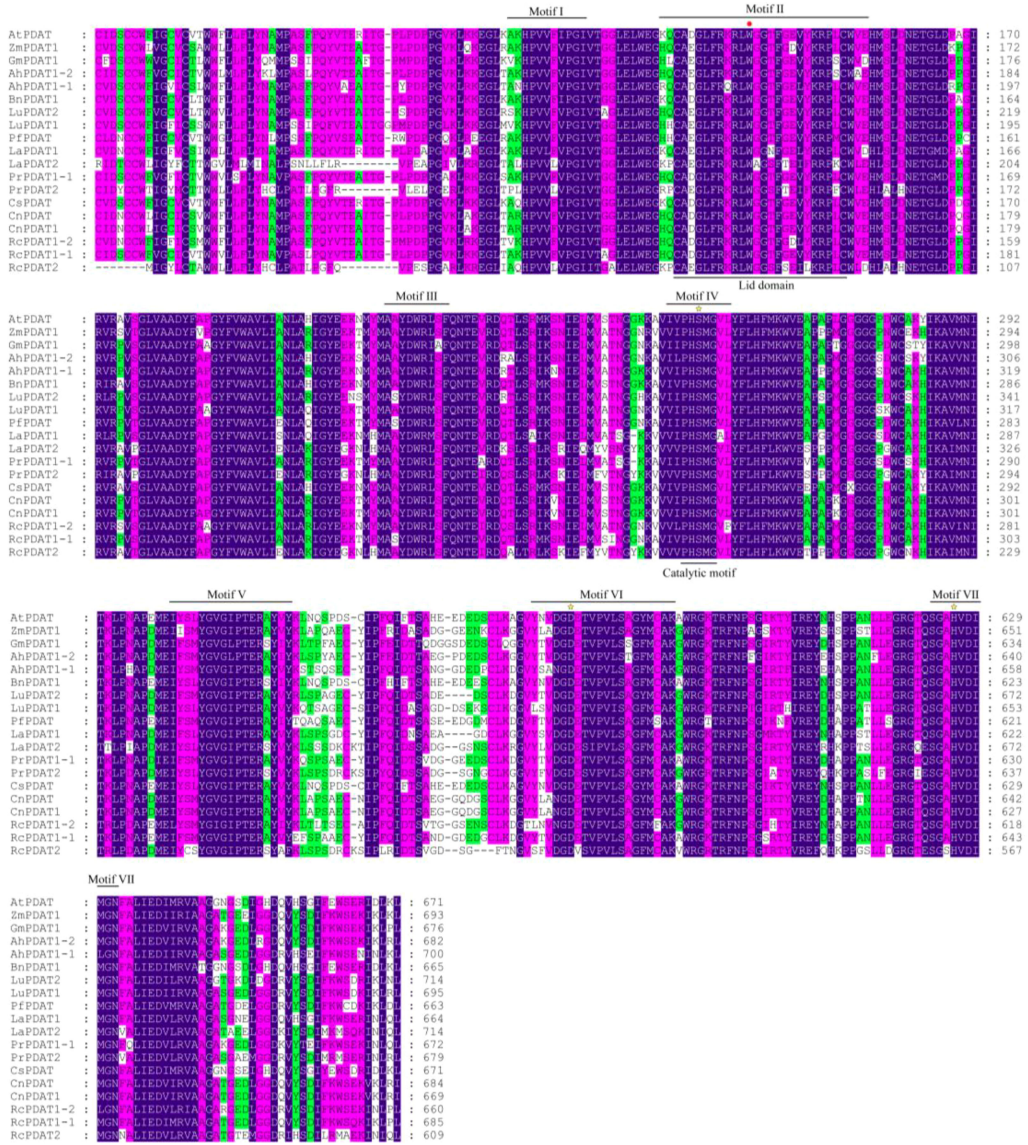
PDAT multi-sequence alignment for select protein sequences and conserved domains in oil-used plants. A red dot represents the Trp residue in motif II, which can involve in removal of fatty acids from the active site; The yellow star refers to the catalytic triplet (Ser-Asp-His). Blue represents 100% similarity, rose red represents more than 80% similarity, and green represents more than 60% similarity. The two-character codes preceding each PDAT indicate the organism of origin are as follows: At, *Arabidopsis thaliana*; Zm, *Zea mays*; Gm, *Glycine max*; Ah, *Arachis hypogaea*; Bn, *Brassica napus*; Lu, *Linum usitatissimum*; Pf, *Perilla frutescens*; La, *Lithospermum arvense*; Cs, *Camelina sativa*; Cn, *Cocos nucifera*; Rc, *Ricinus communis*; Pr, *Paeonia rockii*.

In 2000, Stymne et al. identified PDAT activity in sunflower, castor, and *Crepis* (*Crepis palaestina*) species and cloned the first *PDAT* gene (Dahlqvist et al., 2000). They also discovered that yeast PDAT was homologous to mammalian lecithin:cholesterol acyltransferase, which transfers acyl groups from PC to cholesterol, catalyzing cholesterol ester formation. Among PDATs with validated functions, bioinformatics predictions indicate that PDATs in yeast, castor, *Arabidopsis*, and flax generally contain ER signal peptides. In castor, PDAT1A and PDAT1B are located on the ER, while PDAT2 is localized to the plasma membrane (Kim et al., 2011). In cells, PC and PE are primarily synthesized in the ER, which is also the main site for TAG synthesis and accumulation. ER-localized PDAT directly utilizes PC and PE to synthesize TAG, and newly synthesized TAG can accumulate with TAG produced via other pathways to form oil droplets. However, in studies on *P. rockii*, three proteins, PrPDAT1-1, PrPDAT1-2, and PrPDAT2, have been shown to localize to the ER (Yang et al., 2023). Despite differences in subcellular localization, PDATs across species share many structural similarities. Multiple sequence alignment revealed seven conserved domains (**Figure 3**). Region I, which is highly conserved across all lecithin:cholesterol acyltransferase-like proteins, has an as-yet-undefined function. Region II, also known as the lid region, destabilizes lipid bilayers, facilitating the binding of hydrophobic substrates to the enzyme’s catalytic site. For instance, in *Arabidopsis* PDAT, a tryptophan residue in this region binds free fatty acids released during the decomposition reaction (Ståhl et al., 2004). Region III is highly conserved and likely associated with phospholipid recognition. All PDATs feature a catalytic triad composed of serine, aspartic acid, and histidine (Ser-Asp-His), which is part of the catalytic site. These PDATs share a conserved catalytic motif (G/A/S-X-S-X-G) (Yoon et al., 2012). However, we found that the conserved catalytic motif in oil plants was PHSMG (**Figure 3**).

Differences in the domain structures of the PDAT gene family lead to functional variations among enzymes. The orthologous genes *AtPDAT1* and *AtPDAT2* in *Arabidopsis* exhibit tissue-specific expression. *AtPDAT1* is predominantly expressed in vegetative tissues, while *AtPDAT2* shows significantly higher expression in seeds compared to other tissues (Ståhl et al., 2004; Mhaske et al., 2005; Pan et al., 2015). Phylogenetic analysis has shown that PDAT candidate genes are present in all studied green plants, including algae, lowland plants (mosses and lycophytes), monocots, and eudicots, suggesting that PDATs are evolutionarily conserved (Pan et al., 2015).

In the early stages of PDAT research, its functions were not well understood. PDAT is a major determinant of TAG biosynthesis during exponential growth in yeast, whereas PDAT’s contribution to TAG biosynthesis in *Arabidopsis* seeds is unclear. Although increased PDAT activity has been observed in microsomes prepared from *AtPDAT1*-overexpressing *Arabidopsi*s lines, *AtPDAT1* overexpression does not affect fatty acid or lipid composition (Ståhl et al., 2004). Recent studies, however, have uncovered significant roles of PDAT in the accumulation of specific fatty acids. For example, in *P. ostii* seeds, PDAT expression is correlated with α-linolenic acid accumulation, suggesting that PDATs are responsible for the high α-linolenic acid content in these seeds (Zheng et al., 2022). Homologous *PDAT* overexpression in alfalfa increases the linoleic and α-linolenic acid contents without affecting the total lipid content (Marmon et al., 2017). An increase in α-linolenic acid content is observed when flax *PDAT* is heterologously expressed in *Arabidopsis*. Conversely, heterologous expression of *Arabidopsis PDAT* in rapeseed leads to unexpected reductions in seed oil accumulation (Pan et al., 2013). Additionally, a decrease in the unsaturation of TAG and phosphoglycerides has been observed, indicating that *Arabidopsis*-derived PDAT has subtle but significant effects on rapeseed oil composition (Fenyk et al., 2022). Collectively, these findings suggest that PDATs have a greater influence on the oil composition than the total oil content.

## Discussion

### Substrate preferences of DGATs and PDATs lead to differences in TAG composition

The roles of DGATs and PDATs in triacylglycerol (TAG) synthesis vary among species, and these acyltransferase types also influence the composition and distribution of fatty acids within TAG (Bates and Browse, 2011). In the last decade, significant efforts have been devoted to exploring the substrate specificities of DGATs and PDATs, particularly their preferences in plants that produce unique fatty acids. The primary goal of these studies has been to identify how specific acyltransferases preferentially utilize desired fatty acids to enrich TAG, enabling genetic engineering of traditional oilseed crops to produce special fatty acids that meet industrial and consumer needs (Liu et al., 2012).

As described above, these acyltransferases have been identified in many plants. For example, PDAT in castor has been shown to preferentially assemble ricinoleic acid to form TAG *in vitro*, and PDATs from plants in the *Crepis* genus also catalyze the preferential attachment of the ricinoleic acid acyl group to the glycerol backbone (Dahlqvist et al., 2000). Consistent with *in vitro* findings, castor PDAT overexpression in *Arabidopsis* resulted in substantial hydroxylated fatty acid accumulation in seed oils (Erp et al., 2011; Kim et al., 2011). Similarly, PDAT in flax has been found to preferentially incorporate α-linolenic acid into TAG (Pan et al., 2013). Through the expression of avocado (*Persea americana*) *PaDGAT1* and *PaDGAT2* in yeast cells and tobacco leaves, researchers have found that *PaDGAT1* restores TAG biosynthesis in the yeast quadruple mutant strain H1246 and significantly increases the total lipid content in cells (Behera et al., 2023). Enzyme activity assays have demonstrated that *PaDGAT1* preferentially uses oleic acid over palmitic acid for TAG synthesis. Thus, DGATs and PDATs exhibit distinct preferences for fatty acyl substrates. Different DGATs and PDATs utilize different acyl donor pools, leading to unique impacts on the seed oil composition. For instance, in wild-type *A. thaliana*, *AtDGAT1* can synthetic TAG utilizing PC-derived DAG (2), which can move into oil body to make a bulk PC-derived DAG (3) pool (Regmi et al., 2020). Although AtPDAT1 and AtDGAT2 can synthesize TAG by using DAG (3), these reactions occur rarely under wild-type conditions. However, In the *dgat1–1* mutant, AtPDAT1 is able to use DAG (3). Similar phenomena were found after overexpression of AtDGAT2, GmDGAT2 and RcDGAT2. This difference in DAG pool selection and utilization explains the substrate preferences of DGATs and PDATs for various fatty acyl substrates (Regmi et al., 2020).

**Figure 4:**
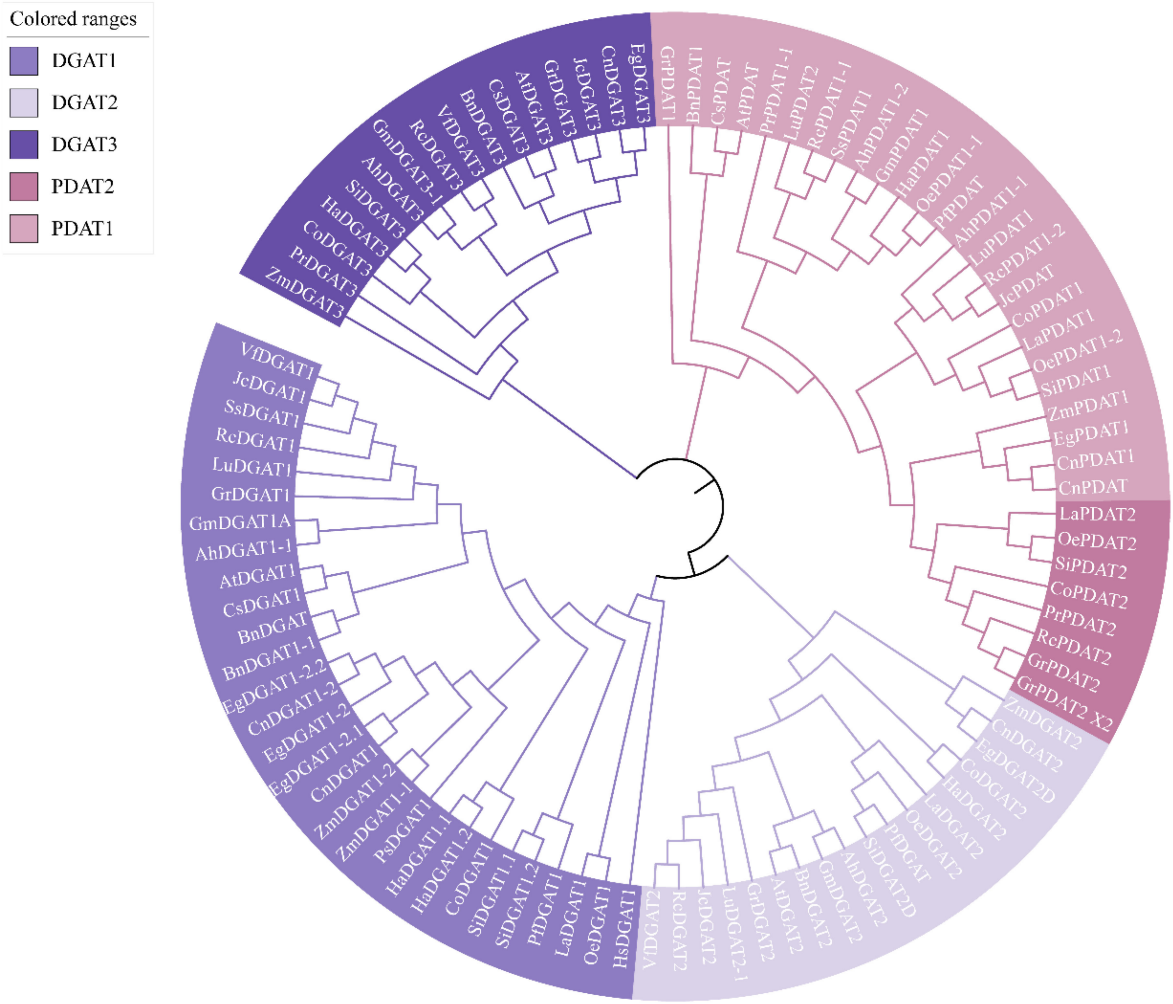
Phylogenetic tree of DGAT and PDAT in oily plants. The two-character codes preceding each DGAT/PDAT indicate the organism of origin are as follows: Hs, *Homo sapiens*; At, *Arabidopsis thaliana*; Zm, *Zea mays*; Gm, *Glycine max*; Si, *Sesamum indicum*; Ah, *Arachis hypogaea*; Ha, *Helianthus annuus*; Bn, *Brassica napus*; Lu, *Linum usitatissimum*; Pf, *Perilla frutescens*; La, *Lithospermum arvense*; Ps, *Paeonia suffruticosa*; Ss, *Sapium sebiferum*; Cs, *Camelina sativa*; Oe, *Olea europaea*; Co, *Camellia oleifera*; Eg, *Elaeis guineensis*; Jc, *Jatropha curcas*; Gr, *Gossypium raimondii*; Cn, *Cocos nucifera*; Rc, *Ricinus communis*; Pr, *Paeonia rockii*; Vf, *Vernicia_fordii*. The sequences of DGAT and PDAT from different species were presented using the nomenclature outlined in **Supplementary Table S1**.

To investigate the relationship between the evolution of DGAT and PDAT in different oil crops and the types of fatty acids they produce, we constructed a phylogenetic tree of 19 oil crops, including 96 DGAT and PDAT sequences (**Figure 4**). From the perspective of differentiation starting point, DGAT1 and DGAT2 share a common evolutionary origin, while DGAT3 is independent of DGAT1 and DGAT2, forming a distinct clade. Similarly, PDAT also clusters separately and is divided into PDAT1 and PDAT2. This indicates that DGAT1/2, DGAT3, and PDAT have undergone distinct evolutionary variations.

Further analysis revealed that in two oil crops, soybean and peanut, which have undergone long-term domestication and selection by humans, their DGAT1, DGAT2, DGAT3, and PDAT1 sequences cluster remarkably well. We propose two possible explanations for this observation: (1) Soybean and peanut both belong to the legume family (Fabaceae) and are closely related phylogenetically, leading to vertical evolution. (2) As traditional oil crops, their oil-related traits, such as oil content and composition, were identified early and subjected to prolonged artificial selection and domestication. Therefore, we hypothesize that long-term human selection may also be a significant factor driving the convergent evolution of DGAT and PDAT in these crops. Additionally, DGAT and PDAT sequences from oil crops capable of synthesizing specialized fatty acids, such as polyunsaturated fatty acids, tend to cluster together. For example, in the DGAT1 branch, Chinese tallow tree, castor bean, and flax can be clustered into one branch; In DGAT2, castor bean and flax can also come together; At the same time, in PDAT1, it was also observed that the PDAT1 of tree peony, Chinese tallow tree and castor bean was clustered into one branch. However, no similar phenomenon was found in DGAT3. Particularly intriguing and noteworthy is the observation that in monocotyledons, the DGAT1 and PDAT1 genes of oil palm and coconut from the Arecaceae family, as well as maize from the Poaceae family, cluster within the same clade, distinctly separated from dicotyledons. In the analysis of DGAT3 genes, maize, as an early-diverging monocot, forms a separate clade, while oil palm and coconut cluster together and group with jatropha and cotton in a distinct branch. Recent phylogenetic and gene structure analyses have revealed that DGAT1, DGAT2, DGAT3, and WS/DGAT genes exhibit distinct evolutionary trajectories in plants, suggesting functional diversification (Turchetto-Zolet et al., 2016). For instance, DGAT3, which is highly expressed during late seed development in soybean and peanut, plays a crucial role in TAG biosynthesis, particularly in the cytosolic pathway (Turchetto-Zolet et al., 2016).

Based on these findings, we speculate that enzymes involved in transporting specialized fatty acids into TAG may be associated with DGAT1, DGAT2, and PDAT1. This is consistent with previous reports. For instance, VfDGAT1 exhibits broad substrate specificity, utilizing 18:2-CoA and 18:3-CoA (Shockey et al., 2006), while transgenic soybean hairy roots expressing GmDGAT2D synthesize higher levels of C18:2-TAG (Chen et al., 2016). Additionally, PDAT has been shown to exhibit high TAG synthesis rates in the presence of acyl chains containing multiple double bonds, epoxy groups, or hydroxyl groups (Sah et al., 2024). The phylogenetic analysis of DGAT and PDAT in different oil crops provides valuable insights into the synthesis of specialized fatty acids and lays a solid foundation for future research on DGAT and PDAT. These studies offer new perspectives for understanding the evolutionary and functional roles of these enzymes in lipid metabolism.

### Contributions of DGATs and PDATs in TAG biosynthesis

In *Saccharomyces cerevisiae*, PDAT and DGAT2 are the primary contributors to TAG biosynthesis, with their relative contributions depending on the yeast’s growth phase (Oelkers et al., 2002). PDAT plays a major role in TAG accumulation during the exponential growth phase, whereas DGAT2 is primarily involved in TAG biosynthesis during the stationary growth phase. Furthermore, no changes in fatty acid content or composition were observed in the seeds of *Arabidopsis AtPDAT1* T-DNA insertion knockout mutants, suggesting functional redundancy between DGAT and PDAT (Mhaske et al., 2005). Subsequent studies have confirmed the overlapping functions of DGAT1 and PDAT1 in *Arabidopsis* TAG biosynthesis. PDAT’s contribution to TAG accumulation in *Arabidopsis* seeds became evident and was only absent when *AtPDAT1* was silenced using RNA interference in a *dgat1* knockout background (Zhang et al., 2009). These results indicate that in the absence of DGAT1 activity, PDAT1 is the primary determinant of TAG biosynthesis in *Arabidopsis* seeds. Similar findings have been reported by Xu et al. who observed significant *AtPDAT1* upregulation in the seeds of *AtDGAT1* mutants, whereas *AtPDAT2* and *AtDGAT2* expression showed only slight changes (Xu et al., 2012). Additionally, other studies have revealed that PDAT contributes to TAG accumulation in *Arabidopsis* leaves (Fan et al., 2013).

Theoretical calculations comparing the relative contributions of DGATs and PDATs to TAG biosynthesis in *Brassica napus* L. indicate that DGATs play a more critical role in this process. Enzymatic selectivity significantly influences the final molecular species composition, with DGATs exhibiting a more pronounced influence on TAG biosynthesis (Woodfield et al., 2018). We propose that incorporating the composition of the acyl-CoA pool, the sn-2 distribution of PC, and the enzyme selectivity coefficient into the model would enhance the accuracy of calculating the contributions of DGAT and PDAT to TAG synthesis. Furthermore, studies on the glyceride flux network dynamics in *Camelina sativa* embryos have revealed that different cellular systems and intermediate lipid pool sizes affect the relative contributions of DGATs and PDATs to TAG synthesis (Pollard and Shachar-Hill, 2022). The influence of enzyme substrate preferences and their interactions with intracellular lipid pools on TAG’s molecular composition provides valuable biotechnological insights for producing seed oils with enhanced oil content and tailored fatty acid profiles.

### Research strategies and applications of DGATs and PDATs in lipid metabolism and bioengineering

The advancement of science has expanded the tools available for studying DGAT and PDAT activities. Two commonly used metrics for assessing enzyme activity are TAG yield and free CoA levels. The most direct and sensitive method for characterizing DGAT biochemical properties involves *in vitro* assays with isotopically labeled substrates, including DAG and acyl-CoA. In animal experiments, permeabilized mammalian cells have been used to measure DGAT activity to study the contributions of DGAT1 and DGAT2 to both the cytoplasmic and luminal sides of the ER membrane (Ganji et al., 2004; Wurie et al., 2011). However, the radioactive nature of isotopes poses safety risks for researchers. Therefore, non-isotopic methods for DGAT activity assays are needed. Innovative approaches have been developed to avoid radioactive substrates. For example, chemical extraction and liquid chromatography-mass spectrometry (LC-MS) were used to study tung tree DGAT functions in 2006 (Shockey et al., 2006). Although effective, this method involves complex sample preparation. Lipophilic fluorescent probes now offer a simpler alternative to qualitative and quantitative enzyme activity analysis by detecting fluorescence intensity. For instance, nitrobenzo-2-oxa-1,3 diazole-labeled fatty acyl-CoA enables *in vitro* activity quantification (McFie and Stone, 2011), while thiol-reactive probes, such as 7-diethylamino-3-(4-maleimidophenyl)-4-methylcoumarin, detect CoA-SH release during acyl-CoA turnover *in vitro* (Cao et al., 2011). In plant and yeast microsomal *in vitro* assays, DGAT prefers endogenous DAG over exogenously added DAG, even when DAG is supplied in excess of a few moles (Lung and Weselake, 2006). This preference complicates *in vitro* DGAT assays, particularly for substrate selectivity studies. In some cases, organic solvents can be used to treat lyophilized microsomes to strip endogenous DAG from membranes, and exogenous DAG is then introduced in the presence of phospholipids, forcing DGAT to rely solely on exogenously added DAG (Liu et al., 2012). To study substrate specificity, researchers often use yeast mutant strains and exogenously supply free fatty acids to identify the DGAT and PDAT preferences of different substrate fatty acids (Shockey et al., 2006; Han et al., 2022).

The application prospects for DGATs and PDATs differ between animals and plants. In plants, DGATs and PDATs are primarily used to increase the lipid content in oil crops and to modify the fatty acid composition of plant oils, aiming to produce higher yields and healthier edible oils. Research findings consistently indicate that DGATs have a greater impact on oil yield in plants, while PDATs play a key role in improving the fatty acid composition of plant oils, particularly that of polyunsaturated fatty acids. In plant research, the future directions and applications of these enzymes can be summarized as follows: (1) Basic research should focus on regulating the metabolic networks and protein interactions of DGAT and PDAT in model plants or oil crops with mature genetic transformation systems to clarify their synergistic functions; (2) Gene editing techniques should be employed to develop novel plant oils tailored to meet human health needs; (3) The metabolic framework of seed storage organelles should be redesigned to optimize oil production. In animals, research on DGATs is more advanced, with a focus on pathways, such as tumor metabolism (Liu et al., 2012); thus, the application prospects for DGATs in animals can be summarized as potential medical treatment targets.

## Conclusion and perspectives

Plant oils are essential for daily life, providing necessary nutrients for human growth and serving as vital industrial raw materials and energy sources. TAG is the main component of storage oils, and its fatty acid composition and content determine the quality and commercial value of these oils. This review summarizes the plant oil types and the molecular processes involved in TAG biosynthesis, with an emphasis on the rate-limiting acyltransferases DGAT and PDAT. The sequence information, structural features, functions, and substrate preferences of these enzyme families are elucidated. Studies have shown that DGAT and PDAT selectively prefer different fatty acid substrates, varying across species and influencing the TAG composition and content in plant oils.

Current research on DGAT and PDAT primarily aims to identify isoenzymes in specific crops and explore their substrate specificity across plants. Although functional redundancy between DGAT and PDAT was proposed over two decades ago, the molecular mechanisms behind this redundancy and the conditions under which DGAT or PDAT becomes the primary factor in lipid synthesis remain poorly understood. Further studies in model organisms, such as *Arabidopsis*, or economically important oil crops, such as soybean, may yield significant insights. Additionally, the complementary roles of DGAT and PDAT under stress conditions or directed modifications remain unexplored. Increasing research on core enzymes in TAG synthesis, particularly PDATs with specific substrate preferences, can provide molecular-level guidance for improving plant oil quality. This may enable targeted modifications in the fatty acid composition and content of TAG, leading to the development of edible oils that meet public health demands.
