# Supplementary material for: Synergistic mechanisms of DGAT and PDAT in shaping triacylglycerol diversity: evolutionary insights and metabolic engineering strategies
Source: Front Plant Sci. 2025 Jul 1;16:1598815. doi: 10.3389/fpls.2025.1598815 (PMC12259708; doi:10.3389/fpls.2025.1598815)
Supplement: Supplementary file 1 [file Table1.doc]

**Supplementary Table 1. Accession numbers of all the protein sequences**

| *Specie* | *Taxa terminologies* | Protein | Database | Access |
| --- | --- | --- | --- | --- |
| *Arabidopsis thaliana* | *At* | AtDGAT1 | NCBI | NP_179535.1 |
|  |  | AtDGAT2 | NCBI | NP_566952.1 |
|  |  | AtDGAT3 | NCBI | NP_175264.3 |
|  |  | AtPDAT | NCBI | NP_196868.1 |
| *Arachis hypogaea* | *Ah* | AhDGAT3 | NCBI | AGT57760.1 |
|  |  | AhDGAT1-1 | NCBI | AGT57761.1 |
|  |  | AhDGAT2 | NCBI | AEO11790.1 |
|  |  | AhPDAT1-2 | NCBI | AID51956.1 |
|  |  | AhPDAT1-1 | NCBI | AID51955.1 |
| *Brassica napus* | *Bn* | BnDGAT1-1 | NCBI | AFM31261.1 |
|  |  | BnDGAT2 | NCBI | XP_013737142.2 |
|  |  | BnDGAT3 | NCBI | XP_013655610.2 |
|  |  | BnDGAT | NCBI | AIU44413.1 |
|  |  | BnPDAT1 | NCBI | XP_048604151.1 |
| *Camelina sativa* | *Cs* | CsDGAT1 | NCBI | AQM52373.1 |
|  |  | CsDGAT3 | NCBI | AQM52372.1 |
|  |  | CsPDAT | NCBI | AQU71887.1 |
| *Camellia oleifera* | *Co* | CoDGAT1 | NCBI | ATQ37961.1 |
|  |  | CoDGAT2 | NCBI | ATQ37962.1 |
|  |  | CoDGAT3 | NCBI | ATQ37963.1 |
|  |  | CoPDAT2 | NCBI | QID89770.1 |
|  |  | CoPDAT1 | NCBI | QID89769.1 |
| *Cocos nucifera* | *Cn* | CnDGAT1 | NCBI | AZZ09170.1 |
|  |  | CnDGAT1-2 | NCBI | KAG1335378.1 |
|  |  | CnDGAT3 | NCBI | KAG1326094.1 |
|  |  | CnDGAT2 | NCBI | KAG1359019.1 |
|  |  | CnPDAT | NCBI | AZZ09174.1 |
|  |  | CnPDAT1 | NCBI | KAG1334251.1 |
| *Elaeis guineensis* | *Eg* | EgDGAT1-2.1 | NCBI | XP_010924968.1 |
|  |  | EgDGAT1-2.2 | NCBI | XP_029119023.1 |
|  |  | EgDGAT1-2 X1 | NCBI | XP_010925471.1 |
|  |  | EgDGAT2D | NCBI | XP_010932136.1 |
|  |  | EgDGAT3 | NCBI | XP_010914783.1 |
|  |  | EgPDAT1 | NCBI | XP_010917136.1 |
| *Glycine max* | *Gm* | GmDGAT1A | NCBI | AMP18197.1 |
|  |  | GmDGAT2 | NCBI | AKR16145.1 |
|  |  | GmDGAT3-1 | NCBI | NP_001412201.1 |
|  |  | GmPDAT1 | NCBI | XP_003528441.1 |
| *Gossypium raimondii* | *Gr* | GrDGAT1 | NCBI | XP_012477685.1 |
|  |  | GrDGAT2 | NCBI | XP_012489339.1 |
|  |  | GrDGAT3 | NCBI | XP_012490255.2 |
|  |  | GrPDAT1 | NCBI | XP_052488101.1 |
|  |  | GrPDAT2 X2 | NCBI | XP_012476314.1 |
|  |  | GrPDAT2 | NCBI | XP_012465460.1 |
| *Helianthus annuus* | *Ha* | HaDGAT1.1 | NCBI | ACD67882.1 |
|  |  | HaDGAT1.2 | NCBI | ABX61081.1 |
|  |  | HaDGAT2 | NCBI | ADT91714.1 |
|  |  | HaDGAT3 | NCBI | XP_022038542.1 |
|  |  | HaPDAT1 | NCBI | XP_022009772.1 |
| *Homo sapiens* | *Hs* | HsDGAT1 | NCBI | NP_036211.2 |
| *Jatropha curcas* | *Jc* | JcDGAT1 | NCBI | ABB84383.1 |
|  |  | JcDGAT2 | NCBI | AFV61670.1 |
|  |  | JcDGAT3 | NCBI | XP_037496303.1 |
|  |  | JcPDAT | NCBI | AEZ56255.1 |
| *Linum usitatissimum* | *Lu* | LuDGAT1 | NCBI | AHA57450.1 |
|  |  | LuDGAT2-1 | NCBI | AHA57445.1 |
|  |  | LuPDAT2 | NCBI | AHA57448.1 |
|  |  | LuPDAT1 | NCBI | AHA57447.1 |
| *Lithospermum arvense* | *La* | LaDGAT1 | NCBI | QPB88922.1 |
|  |  | LaDGAT2 | NCBI | QPB88921.1 |
|  |  | LaPDAT1 | NCBI | UWT60810.1 |
|  |  | LaPDAT2 | NCBI | UWT60811.1 |
| *Olea europaea* | *Oe* | OeDGAT1 | NCBI | AAS01606.1 |
|  |  | OeDGAT2 | NCBI | ADG22608.1 |
|  |  | OePDAT1-1 | NCBI | UVD39197.1 |
|  |  | OePDAT1-2 | NCBI | UVD39198.1 |
|  |  | OePDAT2 | NCBI | UVD39199.1 |
| *Paeonia rockii* | *Pr* | PrDGAT3 | NCBI | WGW06495.1 |
|  |  | PrPDAT1-1 | NCBI | WKY18105.1 |
|  |  | PrPDAT2 | NCBI | WDY36112.1 |
| *Paeonia suffruticosa* | *Ps* | PsDGAT1 | NCBI | AVY53540.1 |
| *Perilla frutescens* | *Pf* | PfDGAT1 | NCBI | AAG23696.1 |
|  |  | PfDGAT | NCBI | KAH6799332.1 |
|  |  | PfPDAT | NCBI | KAH6758187.1 |
| *Ricinus communis* | *Rc* | RcDGAT1 | NCBI | AAR11479.1 |
|  |  | RcDGAT2 | NCBI | AAY16324.1 |
|  |  | RcDGAT3 | NCBI | EEF43203.1 |
|  |  | RcPDAT1-2 | NCBI | NP_001310662.1 |
|  |  | RcPDAT1-1 | NCBI | XP_015576115.1 |
|  |  | RcPDAT2 | NCBI | AEW99983.1 |
| *Sapium sebiferum* | *Ss* | SsDGAT1 | NCBI | APQ41596.1 |
|  |  | SsPDAT1 | NCBI | QQL95683.1 |
| *Sesamum indicum* | *Si* | SiDGAT1.1 | NCBI | AEE37277.1 |
|  |  | SiDGAT1.2 | NCBI | UPO78973.1 |
|  |  | SiDGAT2D | NCBI | XP_011098009.1 |
|  |  | SiDGAT3 | NCBI | XP_011086024.1 |
|  |  | SiPDAT2 | NCBI | UPO78976.1 |
|  |  | SiPDAT1 | NCBI | UPO78975.1 |
| *Vernicia fordii* | *Vf* | VfDGAT3 | NCBI | AGL81309.1 |
|  |  | VfDGAT1 | NCBI | ABC94472.1 |
|  |  | VfDGAT2 | NCBI | ABC94474.1 |
| *Zea mays* | *Zm* | ZmDGAT1-2 | NCBI | ABV91586.1 |
|  |  | ZmDGAT3 | NCBI | AQK41992.1 |
|  |  | ZmDGAT1-1 | NCBI | NP_001349157.1 |
|  |  | ZmDGAT2 | NCBI | AQL03434.1 |
|  |  | ZmPDAT1 | NCBI | ONM22222.1 |
